# The Role of High-output His-bundle Pacing During Atrioventricular Node Ablation

**DOI:** 10.19102/icrm.2022.130705

**Published:** 2022-07-15

**Authors:** Khalil Kanjwal, Wasim Rashid, Asim Kichloo, Abdul Qadir Haji

**Affiliations:** ^1^Department of Electrophysiology, McLaren Greater Lansing Hospital, Lansing, MI, USA; ^2^Shri Kashmir Institute of Medical Sciences, Srinagar, Kashmir, India; ^3^Department of Internal Medicine, Samaritan Medical Center, Watertown, NY, USA; ^4^Department of Internal Medicine, Central Michigan University, Saginaw, MI, USA; ^5^Department of Cardiology, Martinsburg VA Medical Center, Martinsburg, WV, USA

**Keywords:** Anti-arrhythmic, atrial fibrillation, atrioventricular junction, atrioventricular junction ablation, His bundle

## Abstract

Atrioventricular (AV) junction ablation (AVJA) is an acceptable strategy to control the heart rate in atrial fibrillation (AF) with a high procedural success rate. However, a small subset of patients pose a technical challenge with the standard right-sided approach. High-output His-bundle pacing has been shown to help localize the His bundle in a difficult-to-ablate AV junction. We report a case series of patients with difficult-to-ablate AVJA and present strategies to troubleshoot them. In this small series of patients, we found that high-output His pacing can be an effective alternative for successfully localizing the AVJA site. In this series, we also observed that an inability to achieve His capture from the right side can predict failure of ablation using the standard right-sided approach and the consequent need for a left-sided approach.

## Introduction

Atrioventricular (AV) junction (AVJ) ablation (AVJA) is performed in patients with atrial fibrillation (AF) who are not good candidates for a rhythm-control strategy by using either anti-arrhythmic medication or radiofrequency ablation (RFA).^1^ AV node (AVN) ablation is nearly uniformly successful, with a right-sided approach achieving AV block in 95% of patients.^2^ However, a small subset of patients pose a challenge in terms of ablation success. Several techniques have been described to achieve successful ablation in such difficult cases. We present a retrospective case series of such patients and discuss the role of high-output His pacing to achieve ablation success.

## Methods

In this retrospective case series, patients who failed AVN ablation with the standard right-sided approach were identified. All patients suffered drug-refractory rate-uncontrolled permanent AF. These patients had multiple hospitalizations for the control of heart rate and were not candidates for a rhythm-control strategy. In all patients, a single (SR0; Abbott, Chicago, IL, USA) sheath was inserted into the right femoral vein using the modified Seldinger technique under ultrasound guidance. A non-irrigated 8-mm Navistar ablation catheter (Biosense Webster, Diamond Bar, CA, USA) was used to map the His bundle and AVN. Three-dimensional (3D) electroanatomic mapping using the CARTO^®^ system (Biosense Webster) was performed, and the areas with a His signal were tagged on the 3D map. The mapping was performed in the anteroseptal and midseptal areas of the AVJ and base of the right ventricle. The standard right-sided approach was attempted just proximal to the His cloud corresponding to the area of compact AVN. Ablation was performed at 60°C–80°C and a power of 50 W. In patients who failed the standard approach, His pacing was performed using 20 mA at a 2-ms output, and areas where high output resulted in a narrower native-looking QRS were tagged and ablation was performed at those points. Patients who failed to demonstrate His capture were ablated using a retrograde transaortic approach from the left side. The number of ablations attempted at each site varied between 4–6, and each was 45–60 s in duration. The number of sites where ablations were attempted included ≥4 sites in the anteroseptal and midseptal areas of the AVJ. Group 3 had His pacing performed in the beginning, and this group did not have difficult-to-ablate AVNs.

This retrospective study was approved by our institutional review board, and verbal informed consent was obtained from the patients.

## Results

The baseline characteristics of our cohort of 10 patients are presented in **Table 1**.

We divided these 10 patients into 3 groups.

### Group 1

**Figure 1:**
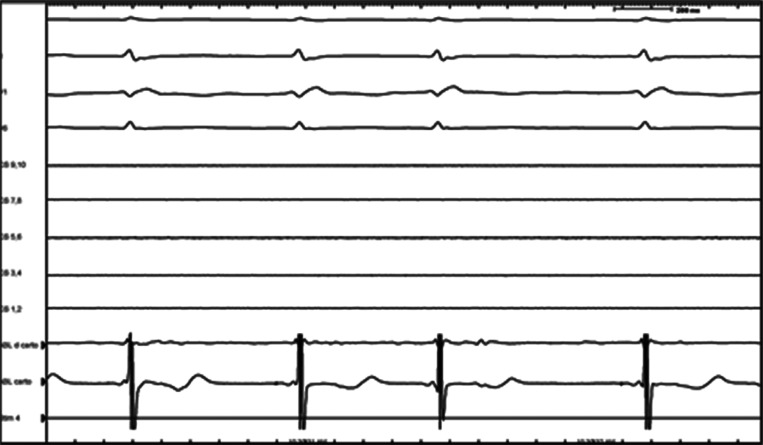
Intracardiac electrogram showing a very small His signal on the second and fourth beats.

**Figure 2:**
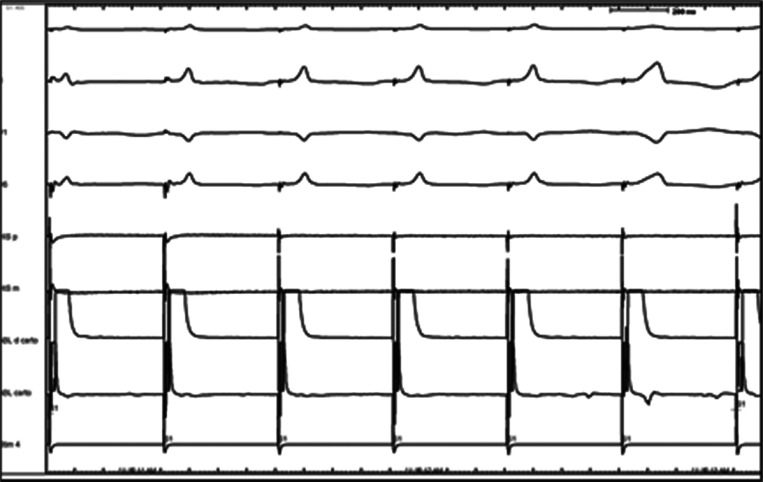
High-output His pacing produces a narrow QRS complex. As the output is decreased, there is a loss of the His capture (wide QRS complex). Subsequent ablation in these areas produced complete heart block.

**Figure 3:**
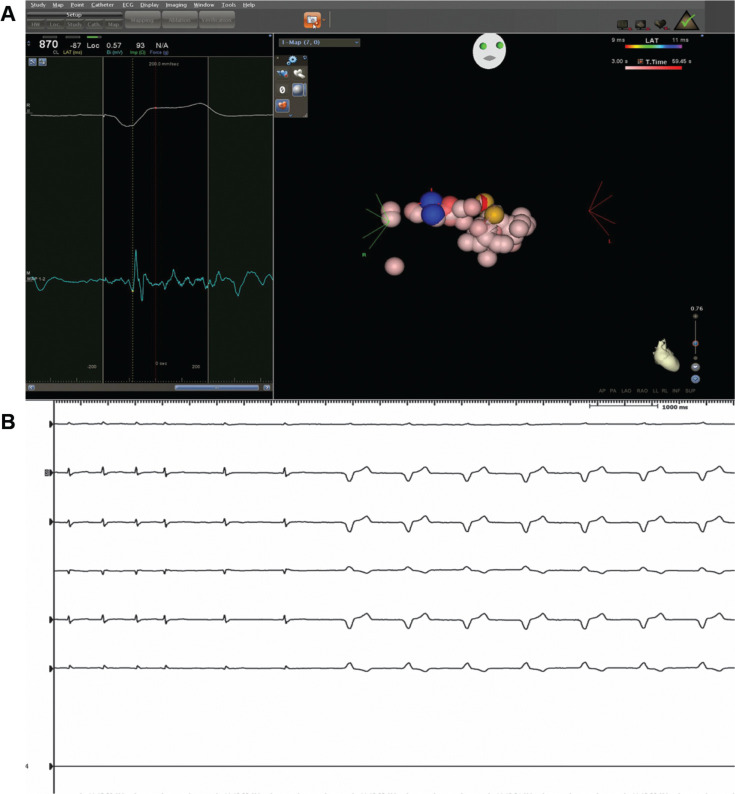
**A and B:** The yellow-tagged areas correspond to the areas where high-output pacing produced direct His capture (narrow QRS). Ablation in the blue-tagged area proximal and posterior to the presumed His area produced complete heart block as is suggested by a paced QRS complex.

This group included 3 patients (aged 68–88 years; all were male) in which the standard right-sided approach was tried first but failed and high-output His pacing was used for localizing the successful ablation site. A non-irrigated 8-mm Navistar ablation catheter was used to map the His bundle and AVN. A small His signal was appreciated on the His electrogram. 3D electroanatomic mapping using the CARTO^®^ system was performed, and the area with a His signal was tagged on the 3D map. Multiple attempts at 60°C–80°C and a power of 50 W failed to achieve heart block. Subsequently, His pacing was performed, and areas with a narrower QRS (His capture) were tagged. Ablation was attempted again in these areas, which resulted in complete heart block. Thus, this group included patients with difficult AVJA, and His-bundle pacing helped in localizing the target site by producing His capture; ablation in these areas was successful from the right side **(Figures 1–3)**.

### Group 2

**Figure 4:**
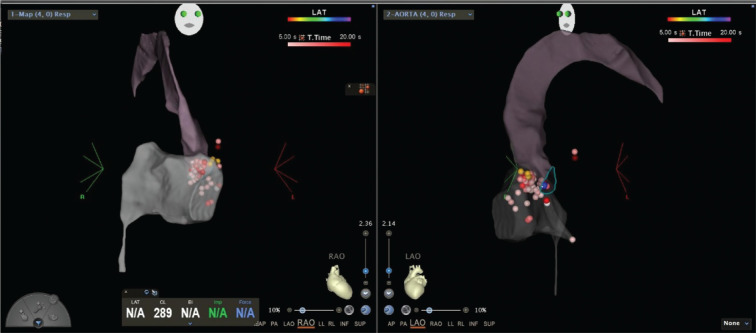
Three-dimensional electroanatomic map showing the right atrium, tricuspid valve, aortic root, and ascending aorta. Multiple ablations from the right side failed to achieve heart block as can be seen by multiple ablation tags from the right side.

**Figure 5:**
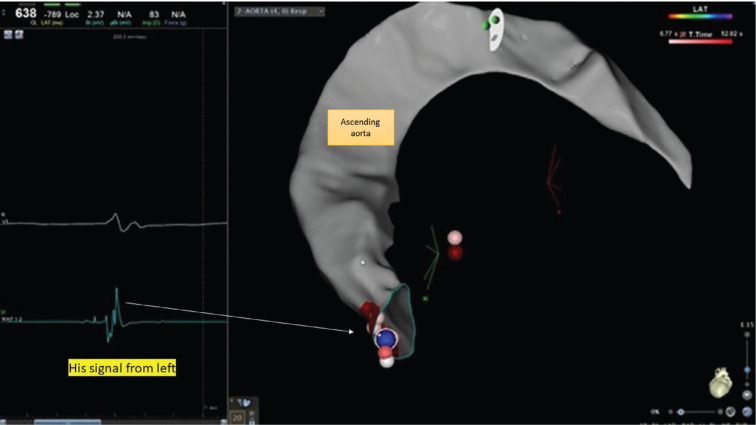
Successful site of ablation below the non-coronary cusp with a His bundle signal of the distal electrode **(left panel)** of the ablation catheter corresponding to the blue dot. A single burn (blue dot) produced complete heart block.

This group included 3 patients (aged 69–76 years; 2 were male) who, after a failed right-sided standard approach, also failed to demonstrate His capture, and a left-sided approach was subsequently used as a bailout for successful ablation. 3D electroanatomic mapping of the septum using the CARTO^®^ system was performed, and the area with a His signal was tagged on the 3D map. Multiple attempts at 60°C–80°C and a power of 50 W failed to achieve heart block. At that point, it was decided to perform high-output pacing to look for capture of the His bundle by producing a narrow QRS complex. However, we were not able to capture the His bundle. Failure of His-bundle capture was likely due to a deeply seated His bundle. It was decided to target the AVJ from the left side. The ablation catheter was advanced to the left ventricle through the right femoral artery. An area beneath the non-coronary cusp was extensively mapped. A single burn in this area with a His signal on the distal electrode of the ablation catheter resulted in instantaneous heart block in all 3 patients **(Figures 4 and 5)**.

### Group 3

In this group of 4 patients (aged 74–86 years; 3 were male), high-output His pacing was performed at the beginning of the case to localize the ablation target. Three patients demonstrated His capture, while 1 did not demonstrate His capture. All 3 patients who demonstrated His capture with high-output pacing were ablated from the right side without any difficulty after a few ablations. The patient in whom we could not demonstrate His capture had an unsuccessful ablation from the right side after multiple (5 attempts, each 1 min in duration at 3 sites) attempts and, subsequently, we performed a left-sided AVJA using a retro-aortic route.

## Discussion

The American College of Cardiology/American Heart Association/Heart Rhythm Society AF practice guidelines indicate that AVJA with permanent ventricular pacing is a reasonable strategy to control the heart rate in AF when pharmacological therapy is inadequate and rhythm control cannot be achieved (class IIa, level of evidence B).^1^ AVJA is a relatively simple procedure, with a standard right-sided approach, achieving AV block in 95% of patients.^2^ However, it can occasionally prove to be a technically challenging procedure. The His signal acts as a surrogate for AVN, and the mapping during AVN ablation usually starts with localizing the His signal, as the current mapping systems are not capable of recording the electrical activity of the AVN. The AVN is localized in the atrial tissue at the apex of the triangle of Koch. The objective of AVJA is to ablate the compact AVN, leaving a stable, ideally junctional escape rhythm. This is usually performed by RFA in the right atrium, typically at a location that displays a large atrial and smaller ventricular electrogram (with an atrial-to-ventricular [A:V] ratio of ≥1) along with a definite His potential on the ablation catheter.^3^ However, the presence of AF often precludes identification of adequate atrial electrograms and makes visualization of the His bundle challenging. Also, the A:V electrogram ratio may be variable in the presence of AF. Unsuccessful ablation attempts can further worsen the situation by creating tissue edema and making it even more difficult to appreciate a good His-bundle signal. Cardioversion to identify the signals has been suggested but practically is not always convenient or feasible. In this context, additional techniques are required for successful AVJA.

We had earlier demonstrated in a case report that, in a difficult-to-ablate AVJ, high-output His pacing can be performed in an attempt to successfully localize and ablate the His bundle.^4^ Subsequently, Kulkarni et al. compared this approach with the standard right-sided approach and showed that His-bundle pacing was an effective technique to identify optimal ablation sites during AVJA and was associated with trends toward fewer RF energy applications and a shorter mean RF time.^5^ Para-Hisian pacing is commonly performed during the electrophysiological study of supraventricular tachycardia to assess whether the retrograde conduction during sinus rhythm occurs through the AVN or a para-Hisian accessory pathway. The His bundle is a deeply insulated structure, and it is difficult to capture it at usual energy outputs. Using a high-output pacing (usually 20 mA at 2 ms), it is possible to directly capture the deeply situated His bundle, which is confirmed by a narrower QRS complex on the paced electrograms.^6^ Thus, high-output pacing can be utilized to map the His-bundle area in a difficult-to-ablate AVJ.

An alternative approach for AVJA is RFA from the left ventricular side through a retrograde transaortic route.^7^ However, this approach carries an increased risk of arterial injury and stroke.

Using a simple maneuver of high-output His-bundle pacing could potentially be very helpful to successfully map and localize the His bundle. This has been substantiated by an earlier report on difficult AVN ablation using the high-output His-bundle pacing and allowing for successful ablation, even though other strategies like using long sheaths and changing to an irrigated ablation catheter were unsuccessful.^5^ Our case series corroborates these findings (group 1). Further, our case series demonstrates that failure to capture His using high-pacing outputs (20 mA at 2 ms) makes it likely that it is situated deep in the septum and may predict the need for ablation from the left side (group 2). The anatomic plausibility of this conclusion may be due to the fact that, if the electric current cannot penetrate and capture the His bundle, it is unlikely that radiofrequency energy will.

In other words, failure of high-output His pacing to capture the His may indicate that the His is deeply situated and may not be amenable to ablation from the right side. However, this presumption needs to be proved in a prospective randomized study.

In the current report, we were able to achieve successful AVJA only after localizing the His bundle with high-output His pacing (group 1). One may argue that His-bundle pacing should have been performed prior to any ablation being attempted and the creation of tissue edema after the failed ablations could interfere with His-bundle capture. This was taken into account in group 3, and we performed His pacing upfront before attempting ablation. Patients who had His-bundle capture underwent successful ablation without any difficulty, and the 1 patient in whom the His bundle could not be captured after multiple attempts at ablation from the right side failed. AVJA was performed from the left side subsequently.

One must keep in mind that, in the AVJ, the ablation target is not the His bundle but the compact AVN. In fact, some studies have stressed the fact that, compared to the AVJA, atrio-nodal input ablation (with a stable escape rhythm, which is closer to normal and also responds to exercise) was associated with a lower mortality rate on long-term follow-up.^8,9^ The His bundle is a surrogate of AVN and alternative approaches should be employed in only difficult-to-ablate cases. Upfront His pacing as a routine to localize the His bundle and replace the standard approach does not seem to be a prudent approach.

### Limitations

There are certain limitations in this case series. This study included only a small group of patients; thus, it was not possible to discern the true incidence/prevalence of patients with a difficult-to-ablate AVJ. Despite this limitation, we believe that failure to capture the His bundle using high-output pacing could be an indicator of unsuccessful or difficult AVJA from the right side; however, a large, randomized study in the future may help to prove these assumptions. We also believe that using this maneuver early on during the procedure may help to avoid the potential complication of continued unsuccessful ablation from the right side and help physicians to make an earlier decision to perform ablation from the left side. The use of this maneuver has the potential to shorten the ablation, fluoroscopy, and total procedure times. Also, in this study, we used an 8-mm catheter only. One may argue that a 4-mm catheter would have had a better resolution in identifying the His signal. However, from our previous experience, in which we used a 3.5-mm irrigated-tip catheter that failed to localize a His signal, we could perform His pacing and avoid changing the catheter after failed AVJAs.^4^ We also used a 3D mapping system in each case, and we want to make it clear that it is not a requirement for standard AVJA.

## Conclusion

High-output His pacing may help to achieve ablation success when the standard right-sided approach fails. Further, it may predict difficult or unsuccessful AVJA from the right side and indicate the need for a left-sided approach for successful ablation.

## Figures and Tables

**Table 1:** Demographics of the Study Population (N = 10)

Age (years)	68–88
White	100%
Male sex	8 (80%)
Comorbidities	
HTN	9 (90%)
Coronary artery disease	6 (60%)
DM	4 (40%)

*Abbreviations:* DM, diabetes mellitus; HTN, hypertension.

## References

[1] January CT, Wann LS, Alpert JS (2014). 2014 AHA/ACC/HRS guideline for the management of patients with atrial fibrillation: executive summary: a report of the American College of Cardiology/American Heart Association Task Force on practice guidelines and the Heart Rhythm Society. J Am Coll Cardiol.

[2] Feld GK (2007). Atrioventricular node modification and ablation for ventricular rate control in atrial fibrillation. Heart Rhythm.

[3] Hoffmayer KS, Scheinman M (2013). Current role of atrioventricular junction (AVJ) ablation. Pacing Clin Electrophysiol.

[4] Kanjwal K, Grubb BP (2016). Utility of high-output his pacing during difficult AV node ablation. An underutilized strategy. Pacing Clin Electrophysiol.

[5] Kulkarni N, Moore C, Pandey A (2017). His-bundle pacing for identifying optimal ablation sites in patients undergoing atrioventricular junction ablation: teaching an old dog a new trick. Pacing Clin Electrophysiol.

[6] Hirao K, Otomo K, Wang X (1996). Para-Hisian pacing: a new method for differentiating retrograde conduction over an accessory AV pathway from conduction over the AV node. Circulation.

[7] Sousa J, El-Atassi R, Rosenheck S, Calkins H, Langberg J, Morady F (1991). Radiofrequency catheter ablation of the atrioventricular junction from the left ventricle. Circulation.

[8] Strohmer B, Hwang C, Peter CT, Chen P-S (2003). Selective atrionodal input ablation for induction of proximal complete heart block with stable junctional escape rhythm in patients with uncontrolled atrial fibrillation. J Interv Card Electrophysiol.

[9] Scherlag BJ, Elkholey K, Stavrakis S, Jackman WM, Po SS (2020). Atrioventricular junctional ablation: the good, the bad, the better. Heart Rhythm O2.

